# Editorial: Role of extracellular vesicles in cancer: implications in immunotherapeutic resistance

**DOI:** 10.3389/fimmu.2025.1689503

**Published:** 2025-09-10

**Authors:** Sheila Spada, Anirban Ganguly

**Affiliations:** ^1^ IRCCS Istituto Nazionale Tumori Regina Elena, UOSD Immunologia e Immunoterapia dei Tumori, Rome, Italy; ^2^ Department of Biochemistry, All India Institute of Medical Sciences, Deoghar, Jharkhand, India

**Keywords:** exosomes, nanoparticles, CD8 T cells, tumor, crosstalk, personalized medicine, onco immunology, dendritic cells

Cancer remains one of the leading causes of mortality worldwide, with therapeutic resistance representing a major challenge to long-term treatment success. Among the several factors contributing to therapy failure, the tumor microenvironment (TME) and intercellular communication have emerged as crucial features (1, 2). Extracellular vesicles (EVs) are critical mediators of cellular crosstalk within the TME. These nano-sized lipid bilayer-bound vesicles shuttle a cargo of bioactive molecules, including proteins, nucleic acids, lipids and multiple microbiota-derived metabolites, that reflects the state of their cells of origin, influencing in turn the hallmarks of cancer such as proliferation, metastasis, angiogenesis, and immune evasion (3–8).

An increasing number of studies have illuminated the pivotal role that EVs play in modulating the immune landscape of tumors, influencing the efficacy of immunotherapies (9). Indeed, EVs can contribute to the development of immunotherapeutic resistance by transferring immunosuppressive molecules or altering antigen presentation pathways. Their ability to reprogram immune cell function and promote an immune-privileged environment presents a significant obstacle to treatments such as immune checkpoint inhibitors and CAR-T cell therapies. Notably, understanding the mechanisms by which EVs modulate immune responses is therefore essential for overcoming resistance and improving clinical outcomes.

This research topic explores the multifaceted role of EVs in cancer, with a specific focus on their role in mediating resistance to immunotherapies. We aim to highlight the molecular mechanisms underpinning EV-mediated immune modulation, evaluate current evidence from preclinical and clinical studies, and discuss the therapeutic potential of targeting EVs to enhance immunotherapy efficacy.

Cancer-derived EVs are potential targets for overcoming resistance to immunotherapy and immune evasion strategies. Ahn et al. illustrated the mechanisms mediated by cancer-derived EVs to modulate the immune system and promote an immunosuppressive TME and a systemic environment that is less prone to effective anticancer immunity. EVs carry several immunomodulatory molecules, including PD-L1, TGF-β, and FasL, affecting the functions of dendritic, T and NK cells, contributing to the failure of immuno-checkpoint blockade and CAR-T cell therapies. Being involved in intercellular communications, EVs favor metastasis and targeted therapy resistance. Therefore, targeting cancer-derived EVs might counteract EV-mediated immunosuppression and open avenues to future directions for enhancing cancer immunotherapy. Accordingly, Morini et al. described the relevant roles of tumor-derived EVs (TEVs) on immune cells, by inactivating CD8+ T cells, inducing polarization of macrophages towards M2 phenotype, inhibiting the activation and inducing the apoptosis of NK cells as well as inhibiting the secretory function of dendritic cells. These effects crucially impact immunotherapy-based treatments in neuroblastoma. In hepatocellular carcinoma (HCC), EVs derived from cancer cells as well as tumor-associated macrophages, by transferring cellular components of the TME act as mediators of immune response by modulating the macrophage polarization, thus impacting tumor growth, metastasis, glycolysis and drug resistance, as reported by Xu et al. The multinetwork regulatory mechanisms of EVs in HCC pathogenesis was elucidated by Yuan et al. They detailed the crucial roles of EVs in progression and cancer cell proliferation in HCC mediated via the modulation of PI3K/AKT and Wnt/β-catenin signaling pathways accompanied with a concomitant reprogramming of the TME cellular composition and functioning and the enhancement of malignant behaviors. The biologically potent molecular libraries comprising of proteins, lipids and nucleic acid components carried by the EVs result in systematic integration to very complex intercellular communication networks leading to a characteristic metabolic-immune cross-regulatory network regulated at multi-dimensional levels and orchestrated by cascading hubs of signal transduction pathways, highlighting the translational value of EVs as precision medicine targets. Moreover, the role of EVs in hepatoma progression was studied by Sun at al. demonstrating that hepatic stellate cells-derived EVs transfer high levels of miR-27a-3p inducing M2 macrophage polarization and promoting hepatoma progression. The phenomenon of drug resistance in cancer cells is often acquired by cell-to-cell communication mediated by the EVs, which transport the cargo of miRNAs and efflux transporters to the previously chemo-sensitive cells. This process was further highlighted by Santos et al., who elucidated the role of transcription changes modulated by EVs derived from tamoxifen- and doxorubicin-resistant breast cancer cells in sensitive cells and also studied how these EVs induced increased drug resistance with a concomitant inhibition of apoptotic pathways, resulting in increased survivability of the sensitive cells. Aligned with prior research, Wang et al. reviewed the implications of EVs in therapeutic resistance in a spectrum of neoplasms, from gynecological and breast cancer, prostate cancer, to lung and colorectal cancer. EVs drive immune evasion and therapy resistance through diverse mechanisms, including the transfer of immunosuppressive molecules (TGF-β, adenosine), antigen masking and immune evasion shedding tumor associated antigens (HER2, MUC1), that affect immune cell function and enhance tumor survival. Recent research by Liu et al. reviewed currently available scientific literature to understand the role of EVs in augmenting immunotherapy in gastric carcinoma (GC) along with highlighting the potential of EVs as therapeutic delivery carriers. These EVs provide a medium for communication between GC cells and different types of cells within the TME. In GC, EVs also act as important biomarkers for prognosis, diagnosis and treatment and act as promising vectors for targeted drug delivery rendering them very useful targets for enhancing success for immunotherapy.

Understanding the mechanisms by which EVs contribute to immunotherapeutic resistance could unlock opportunities for innovative therapeutic strategies, including the development of EV-targeted therapies or their use as biomarkers to predict and monitor responses to immunotherapy. Continued research in this field holds great promise for overcoming current therapeutic challenges and advancing the development of more precise and personalized cancer treatments.
