# Correction: Editorial: Role of extracellular vesicles in cancer: implications in immunotherapeutic resistance

**DOI:** 10.3389/fimmu.2025.1720080

**Published:** 2025-10-17

**Authors:** Sheila Spada, Anirban Ganguly

**Affiliations:** ^1^ IRCCS Istituto Nazionale Tumori Regina Elena, UOSD Immunologia e Immunoterapia dei Tumori, Rome, Italy; ^2^ Department of Biochemistry, All India Institute of Medical Sciences, Deoghar, Jharkhand, India

**Keywords:** exosomes, nanoparticles, CD8 T cells, tumor, crosstalk, personalized medicine, onco immunology, dendritic cells

Affiliation “IRCCS Istituto Nazionale Tumori Regina Elena, UOSD Immunologia e Immunoterapia dei Tumori, Rome, Italy” was erroneously given as “Tumor Immunology and Immunotherapy Unit, Regina Elena National Cancer Institute, Rome, Italy”.

The original version of this article has been updated.

